# Physical activity across the lifespan and liver cancer incidence in the NIH‐AARP Diet and Health Study cohort

**DOI:** 10.1002/cam4.1343

**Published:** 2018-03-13

**Authors:** Hannah Arem, Erikka Loftfield, Pedro F. Saint‐Maurice, Neal D. Freedman, Charles E. Matthews

**Affiliations:** ^1^ Department of Epidemiology and Biostatistics Milken Institute School of Public Health George Washington University Washington District of Columbia; ^2^ GW Cancer Center Washington District of Columbia; ^3^ Division of Cancer Epidemiology and Genetics National Cancer Institute Bethesda Maryland

**Keywords:** Epidemiology and prevention, life course, liver cancer, physical activity

## Abstract

While liver cancer rates in the United States are increasing, 5‐year survival is only 17.6%, underscoring the importance of prevention. Physical activity has been associated with lower risk of developing liver cancer, but most studies assess physical activity only at a single point in time, often in midlife. We utilized physical activity data from 296,661 men and women in the NIH‐AARP Diet and Health Study cohort to test whether physical activity patterns over the life course could elucidate the importance of timing of physical activity on liver cancer risk. We used group modeling of longitudinal data to create physical activity trajectories using four time points across the life course from teenage years through middle age, identifying seven distinct trajectories. We then used Cox proportional hazards regression to assess the association between the physical activity trajectories and risk of hepatocellular carcinoma, the most common type of liver cancer. We found that, in adjusted analyses, compared to those with consistently low physical activity patterns, those who *maintained* activity levels over time had a 26–36% lower risk of liver cancer and those who *increased* physical activity over time had no associations with risk, while those who *decreased* activity over time had a nonsignificantly higher risk of liver cancer. Our results suggest that sustained physical activity is associated with lower risk of hepatocellular carcinoma, while increasing physical activity later in life may not yield the same benefit. Future research with larger sample sizes and more detailed data on dose and timing of physical activity may continue to yield insight into this association between physical activity and liver cancer risk.

## Introduction

In the United States, from 2003 to 2012, rates of incident liver cancer rose 38% 1. The cause of increasing liver cancer rates has been largely attributed to viral hepatitis C (accounting for an estimated 50% of incidence), as well as to increases in prevalence of diabetes, obesity, and fatty liver disease 2. Furthermore, there will be an estimated 28,920 deaths due to liver cancer in 2017, with only 17.6% percent of those diagnosed surviving 5 years 3. The strongest known risk factors for developing hepatocellular carcinoma (HCC), the most common form of liver cancer, include cirrhosis (most often caused by alcohol abuse) and chronic hepatitis B or C viral infections 4. Other known risk factors include aflatoxin intake, tobacco smoking, obesity, and diabetes 4, 5, 6, 7. Higher coffee intake has shown an inverse association with liver cancer risk 5. The upward trend in incidence and rapid fatality highlights the importance of liver cancer prevention. Physical activity is one such promising means of liver cancer risk reduction.

The 2015 Continuous Update Project on liver cancer from the World Cancer Research Fund International found a “limited–suggestive” level of evidence that physical activity decreases risk of liver cancer 5. Previous studies have shown associations between physical activity and lower risk of liver cancer incidence and death, although statistical significance of results varies by the study 8, 9, 10. The previous study in this National Institutes of Health (NIH)‐AARP Diet and Health Study cohort showed a 36% lower liver cancer risk comparing high to low physical activity levels in the year prior to baseline questionnaire (HR = 0.64, 95% CI 0.49–0.84 for 5+ times/week compared to never/rare exercise) 11; the largest pooled analysis of prospective studies to date (including NIH‐AARP) with a total of 1384 cases showed a 27% lower liver cancer risk (95% CI 0.55–0.98) comparing high versus low exercisers. However, we do not know much about the timing of physical activity over the life course and whether it is most important that one was physically active in midlife, or whether there is any cumulative benefit to being active also in adolescence and early adulthood. Most of the published studies used self‐reported physical activity at only a single point in time, most commonly in midlife, which may not account for the long‐term maintenance of physical activity patterns or increases and decreases in activity levels over time that may affect risk for obesity and poor metabolic health 12. Some recent studies suggest that physical activity causes epigenetic changes that relate to the development of metabolic disease and perhaps cancer, showing that physical activity was associated with higher methylation in peripheral blood lymphocytes in a class of repeated sequences in the human genome 13.

We hypothesized that assessment physical activity, as measured over the life course as trajectories 14, would provide additional information on how maintenance of and changes in activity levels over time might be associated with liver cancer risk. While recent physical activity may have the strongest effect on metabolic markers, we predicted that, for a disease with a long latency such as liver cancer, sustained physical activity over many years would show the strongest associations with lower risk.

## Methods

### Study population

The NIH‐AARP Diet and Health Study cohort has been previously described 15. In short, the NIH‐AARP cohort included 566,398 AARP members (aged 50–71 years) who completed a mailed baseline questionnaire in 1995–1996. In 1996–1997, an additional risk factor questionnaire (RFQ) including additional questions about participation in physical activity was mailed to participants who did not have self‐reported cancer of the colon, breast, or prostate at the time of the baseline questionnaire (response rate 67%). Participants resided in six states (California, Florida, Pennsylvania, New Jersey, North Carolina, or Louisiana) or two metropolitan areas (Atlanta, Georgia or Detroit, Michigan). Of the 334,905 men and women who completed the baseline and risk factor questionnaires, individuals were excluded if the questionnaire was completed by proxy respondent (*n* = 10,383), reported a prevalent cancer (self‐reported or registry confirmed) at the time of RFQ (*n* = 18,971), anyone who died prior to their questionnaire being scanned (*n* = 26), and anyone who was missing data on the main exposure of interest (*n* = 8864). These exclusions resulted in an analytic sample size of *N* = 296,661. Follow‐up time was calculated from completion of the RFQ until (any) liver cancer diagnosis, death, or end of study follow‐up (12/31/2011). Average follow‐up time was 13.1 years.

The NIH‐AARP study was approved by the Special Studies Institutional Review Board of the US National Cancer Institute, and all participants provided informed consent by completing and returning the baseline questionnaire.

### Liver cancer incidence

Information on date of cancer diagnosis was gathered from cancer registries 15. HCC is the most common type of primary liver cancer and accounts for nearly all cases in the NIH‐AARP cohort. Thus, our primary outcome was defined as HCC (C22.0) with morphology codes of 8000, 8010, 8140, 8170, 8171, 8175, and 8190. As a sensitivity analysis, we further restricted classification of participants as having HCC to those who had specific morphology of HCC (8170–8175) 16. A comparison of cancer registry case ascertainment with SEER estimates and self‐reporting determined that more than 90% of incident cancers across the state registries were identified 17.

### Exposure assessment

Information on demographic characteristics, diet, and reproductive and medical history were collected from the 1994–1995 baseline questionnaire, while the 1995–1996 RFQ queried about recreational physical activity at four distinct points in time: ages 15–18, 19–29, 35–39, and the past 10 years. Participants were asked “How often did you participate in moderate‐to‐vigorous activities” between specific age ranges. Examples of moderate‐intensity to vigorous‐intensity activities such as tennis, weight lifting, biking, swimming, and fast walking were listed. Participants chose from response categories of none, rarely, <1, 1–3, 4–7, and >7 h/week.

### Statistical analysis

We used means and frequency tables to examine the distribution of baseline characteristics by distinct physical activity trajectories. For the main analysis, we used latent class trajectory models to identify trajectories of physical activity at four points in time (SAS Proc Traj, Cary, NC) 18. This method has been previously applied to BMI and outcomes such as diabetes and mortality 19, 20. As choosing the optimal number of trajectories is an iterative process, we chose to assess 3–8 groups a priori and used Bayesian information criteria (BIC) to select the final number of groups, while maintaining a >5% of the population in each group to have reasonable precision in our analysis 14, 21. The BIC is based on the likelihood function; whereby, the lowest value is best. Linear, quadratic, and cubic trajectories were considered.

While we tried to maintain detailed categories where possible, we used the recommended PA minimum of 150 min per week of moderate‐intensity activity, equivalent to 2.5 h/week 22, as our cutoff separating “low” from “high” physical activity levels in the results.

Cox proportional hazards models were used to estimate hazard ratios (HR) and 95% confidence intervals (CI) in SAS version 9.4 (SAS Proc Phreg). The underlying time metric was calculated from age at risk factor questionnaire to age at cancer diagnosis or end of follow‐up, whichever occurred first. We evaluated the proportional hazards assumption by modeling interaction terms of the trajectory groups with follow‐up time; no deviations were observed (all *P*‐values >0.1).

A priori, we decided to include coffee and alcohol consumption in the models as these have been associated with liver cancer in previous studies in this cohort and in the literature. We also tested remaining variables in Table 1 as potential confounders and deemed them as such if they changed parameter estimates by >10%. BMI was calculated as kg/m^2^ using baseline self‐reported height and weight. Physical activity HR estimates were modeled with and without adjustment for BMI and diabetes to address the possibility that each may be in the causal pathway between physical activity and liver cancer risk.

**Table 1:** Demographics and characteristics by physical activity trajectory at baseline

PA patterns over time	Trajectory 1	Referent	Trajectory 3	Trajectory 4	Trajectory 5	Trajectory 6	Trajectory 7
	Low age 15–18 then maintain high	Consistently low	Gradual increase from low to high	Consistently high	Starting high, decreasing after age 39	U‐shaped (met physical activity recommendations)	Gradual decrease from high to low
Demographic							
Age at RFQ (mean, sd)	63.1 (5.3)	62.7 (5.4)	63.0 (5.4)	63.0 (5.2)	62.9 (5.2)	62.9 (5.2)	62.1 (5.4)
Body mass index, kg/m^2^ (mean, sd)	26.3 (4.9)	27.3 (5.4)	25.6 (4.4)	26.4 (4.5)	28.2 (5.5)	26.0 (3.9)	27.8 (5.2)
Sex, %							
Male	34.3	51.3	54.6	60.6	60.8	75.3	69.3
Female	65.7	48.7	45.4	39.4	39.3	24.7	30.8
Married or living as married	60.1	62.9	67.3	70.6	67.2	77.4	72.9
Race (% non‐Hispanic White)	93.3	91.2	93.1	93.8	92.6	94.1	92.2
Education, %							
Less than high school	30.6	28.6	22.4	22.2	20.5	14.0	18.9
Completed high school or posthigh school technical training	35.1	31.5	30.4	34.1	37.1	28.6	33.3
College or greater	31.9	37.4	44.9	41.2	39.8	55.5	45.7
Alcohol intake, g/day (mean, sd)	10.6 (30.2)	11.4 (35.3)	12.2 (31.4)	13.8 (35.8)	14.3 (40.9)	14.9 (33.5)	14.1 (39.0)
Red meat intake, g/day (mean, sd)	21.9 (25.0)	25.1 (30.0)	20.3 (23.8)	27.1 (29.7)	30.4 (32.0)	23.7 (25.9)	29.6 (30.4)
Coffee intake, cups/day (mean, sd)	2.0 (1.6)	1.9 (1.6)	1.9 (1.6)	2.0 (1.6)	2.0 (1.6)	2.0 (1.6)	2.0 (1.6)
Smoking, %							
Current	11.0	11.6	6.4	10.7	14.5	6.2	12.3
Former	45.3	46.3	53.6	49.6	50.9	58.3	51.3
Never	40.8	38.6	36.6	36.4	31.5	32.3	33.4
History of diabetes, %	6.4	9.8	6.8	6.6	10.9	7.0	10.4
Health status, %							
Excellent/very good	56.9	47.1	64.5	61.9	43.2	66.1	48.0
Good	32.6	37.9	27.9	29.4	37.7	27.1	37.6
Fair/poor	9.4	13.7	6.5	7.5	17.6	5.7	13.2
HCC cases, *n*	23	101	40	126	93	23	91

RFQ, Risk factor questionnaire; HCC, hepatocellular carcinoma.

Our final model (Model 2) included sex (male and female), race (non‐Hispanic White, African American, Other, and Missing), coffee intake (nondrinkers, ≤1 c/day, 2–3 c/day, 4–5 c/day, and 6+ c/day), alcohol intake (nondrinkers, ≤1 drink/day, 1 to <3 drinks/day, and ≥3 c/day), and smoking (never, former, and current). Finer adjustment for smoking including time since quitting and pack‐years did not affect parameter estimates (data not shown); thus, never, former, and current were used in models. An additional model (Model 3) was run including BMI (18.5 to <25, 25 to <30, 30 to <35, 35+ kg/m^2^, and missing) and diabetes (yes/no), but main finding presented does not include these factors as they are likely in the causal pathway. We ran additional analyses of physical activity reported over the prior 10 years to assess only most recent activity.

In sensitivity analyses we restricted analyses to those who had no history of diabetes. We also ran models limited to those who reported excellent, very good, or good health to lessen the likelihood of reverse causation. We excluded individuals who developed cancer within 2 years of completing the questionnaire to assess potential latent disease. We were unable to stratify by sex or BMI due to an insufficient number of cases in each trajectory in subsets of the population. All statistical tests were two‐sided, with *P*‐values <0.05 considered statistically significant.

## Results

**Figure 1:**
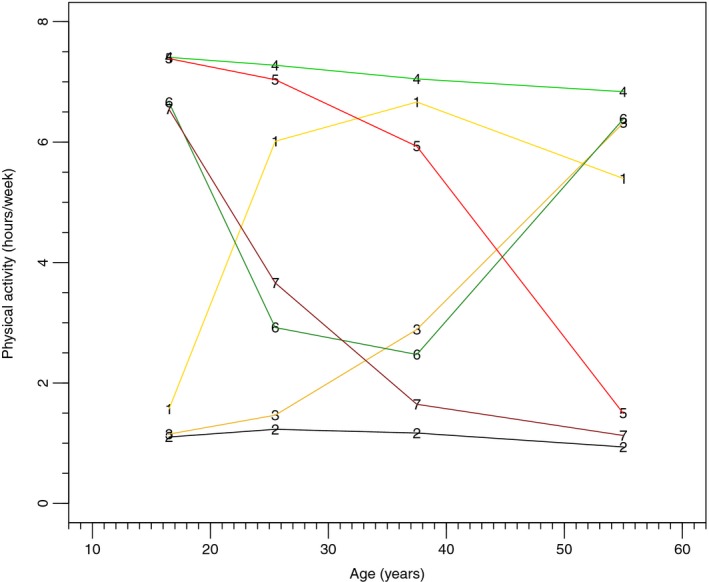
Trajectories of physical activity over the life course. Maintainers are shown in green, increasers are shown in yellow, and decreasers are shown in red.

We identified *n* = 497 incident HCC cases using the broader HCC definition and *n* = 417 cases using the more restrictive definition. We observed seven unique physical activity trajectories in this cohort, which we can also group into three broader patterns: maintainers, decreasers, and increasers. The seven trajectories were assessed separately, but are also grouped for interpretation: *Maintainers* were those who reported activity levels below the recommended minimum (referent, group 2, ~1 h/week) and those who had consistently high activity levels (group 4, ≥7 h/week), as well as those with a U‐shaped pattern (group 6, ≥3 h/week at lowest point); *increasers* were those who reported <2 h/week activity during the teenage years and then ≥6 h/week activity through the 20s and later in life (group 1) and those who reported a gradual increase in activity from <2 to 6 h/week beginning in the 30s (group 3); *decreasers* included those who reported 7 h/week activity in the teenage years through the 30s and then a decrease to <2 h/week activity after age 39 (group 5) and those who reported gradual decrease from >6 to 1 h/week activity (group 7; Fig. 1). We considered the U‐shaped pattern in the maintainer category because, even at the lowest point, the estimated physical activity levels were above 2.5 h/week, which meets the recommended PA minimum of 150 min weekly.

Comparing demographics across these seven trajectories, we found that a greater percentage of men than women reported physical activity maintenance (meeting physical activity recommendations) and a greater percentage reported decreasing physical activity over time; Table 1). Women reported more of an increase in activity levels over the life course, with low activity in teenage years followed by high activity levels. Body mass index was slightly higher among those who reported lowest activity levels most recently. In adjusted analyses, compared to those with consistently low physical activity patterns, among those who *maintained* consistently high physical activity there was a 26% lower risk of liver cancer (HR = 0.74, 95% CI 0.57–0.96) and those who reported a U‐shaped physical activity pattern had a nonstatistically significant 36% lower risk of liver cancer (0.64, 95% CI 0.41–1.01). Those who *increased* physical activity over time had more variable estimates, which were not statistically significant, but were in the inverse direction compared to those who reported low activity over time (HR_active beginning in 20s_ = 0.79, 95% CI 0.50–1.25; HR_active beginning in 30s_ = 0.94, 95% CI 0.65–1.36). Those who *decreased* activity over time showed nonsignificant higher risks of liver cancer (HR_decreasing activity after age 39_ = 1.27, 95% CI 0.96–1.68; HR_steady decrease over time_ = 1.11, 95% CI 0.84–1.48; Table 2).

**Table 2:** Hazard ratios for lifetime physical activity trajectories with liver cancer incidence

	Referent	Trajectory 4	Trajectory 6	Trajectory 3	Trajectory 1	Trajectory 5	Trajectory 7
	Maintainers			Increasers		Decreasers	
	Consistently low	Consistently high	U‐shaped (met activity recommendations)	Low through the 20s, then steady increase beginning in 30s	Low in teens, then maintain high beginning in 20s	High in teens through 30s decreasing after age 39	Steady decrease over time
HCC cases, *n*	101	126	23	40	23	93	91
Model 1	1.00	0.70 (0.54–0.90)	0.60 (0.38–0.94)	0.88 (0.61–1.27)	0.75 (0.48–1.19)	1.28 (0.96–1.69)	1.10 (0.83–1.46)
Model 2	1.00	0.74 (0.57–0.96)	0.64 (0.41–1.01)	0.94 (0.65–1.36)	0.79 (0.50–1.25)	1.27 (0.96–1.68)	1.11 (0.84–1.48)
Model 3	1.00	0.81 (0.61–1.05)	0.71 (0.45–1.13)	1.06 (0.73–1.53)	0.85 (0.54–1.34)	1.18 (0.89–1.57)	1.08 (0.81–1.43)

Model 1. Adjusted for sex, with age as the underlying time metric.

Model 2. Adjusted for sex, with age as the underlying time metric. Also adjusted for race, coffee intake, alcohol intake, smoking history, and no diabetes or BMI.

Model 3. Adjusted for sex, with age as the underlying time metric. Also adjusted for race, coffee intake, alcohol intake, race, smoking history, diabetes, and BMI.

Sensitivity analyses (Table S1) among those with no history of diabetes attenuated associations; directions of associations and magnitudes were similar for activity maintainers (high and U‐shaped) compared to consistently low, while there was still evidence of a higher risk of liver cancer among the decreasers. For the increasers, those who maintained high activity in the 20s and beyond had an attenuated inverse, nonsignificant HR = 0.91 (0.55–1.52), while those who did not start increasing activity until the 30s had a nonsignificant HR = 1.12, 0.74–1.68) compared to those who had consistently low activity patterns. Excluding those who reported fair or poor health and those with <2 years of follow‐up did not show patterns different from our main findings; adding BMI at age 18 to the models also did not change parameter estimates.

In additional sensitivity analyses using a more stringent definition of HCC with *n* = 417 cases, patterns were similar; whereby, the direction of association, while not statistically significant, was inverse for those who reported U‐shaped physical activity trajectory and consistently high physical activity patterns over time (Table S1).

When we assessed the most recently reported moderate‐intensity to vigorous‐intensity physical activity levels (which queried about general habits over the 10 years prior to study baseline) and liver cancer risk (Table 3), we found that compared to those reporting never or rare physical activity, after full adjustment (including BMI and diabetes), there was a ~30% lower risk of liver cancer among those reporting 4+ h/week (HR_4–7 h/week_ = 0.68, 95% CI 0.52–0.90; HR_>7 h/week_ = 0.70, 95% CI 0.53–0.93), with a significant dose– response relationship (*P*‐trend = 0.007).

**Table 3:** PA and liver cancer incidence by physical activity over the prior 10 years

	Moderate‐intensity to vigorous‐intensity physical activity					*P*‐value
	Never/rare	<1 h/week	1–3 h/week	4–7 h/week	>7 h/week	
Model 1	1.0	0.69 (0.50–0.96)	0.70 (0.54–0.90)	0.51 (0.39–0.67)	0.51 (0.38–0.66)	<0.001
Model 2	1.0	0.73 (0.53–1.02)	0.76 (0.59–0.99)	0.57 (0.44–0.75)	0.57 (0.43–0.75)	<0.001
Model 3	1.0	0.78 (0.56–1.08)	0.85 (0.66–1.10)	0.68 (0.52–0.90)	0.70 (0.53–0.93)	0.007

Model 1. Adjusted for sex, with age as the underlying time metric.

Model 2. Adjusted for sex, with age as the underlying time metric. Also adjusted for race (non‐Hispanic White, Black, and Other), coffee intake (nondrinkers, ≤1 c/day, 2–3 c/day, 4–5 c/day, and 6+ c/day), alcohol intake (nondrinkers, ≤1 drink/day, 1 to <3 drinks/day, and ≥3 c/day), and smoking history (never, former, and current).

Model 3. Adjusted for factors in Model 2, as well as diabetes (yes/no), and BMI (18.5 to <25, 25 to <30, 30 to <35, and 35+ kg/m^2^).

## Discussion

After adjusting for potential confounders, we found that, compared to those reporting consistently low physical activity over the life course, those who maintained physical activity over time had a lower risk of liver cancer. While not statistically significant, those who decreased physical activity over time had a suggested higher risk of liver cancer and those who increased activity had stronger protective associations if they began exercising sooner compared to the low exercisers. Although statistical power was limited, these findings suggest that activity only in adolescence and decreasing activity patterns over time are not associated with lower risk of liver cancer, while maintaining high activity and increasing activity in early adulthood may have a protective association. Thus, our results suggest that measuring physical activity only in the prior 10 years may obscure higher risk from lower risk groups depending on when activity was initiated. Although for a different cancer site, this finding of the strongest inverse associations among those who remain active is consistent with a previous evidence for colon cancer, showing that vigorous physical activity at multiple time periods is associated with lower cancer risk 23.

The body of literature on physical activity and liver cancer incidence is summarized in a 2016 pooled analysis of 10 studies with 1384 cases that reported a HR = 0.73 (95% CI 0.55–0.98) comparing high to low exercisers 24. The previously published NIH‐AARP study on physical activity and liver cancer incidence reported a HR = 0.64, 95% CI 0.49–0.84 for 5+ times/week compared to never/rare exercise 11. This finding is slightly stronger, but similar to what we observed with a more detailed physical activity question and an additional 5‐year follow‐up. Our findings on physical activity across the life course offer additional insight, suggesting the importance of regular physical activity over a sustained period of time in relation to risk of liver cancer. Furthermore, the observed patterns in our study suggest that physical activity in earlier life was not associated with lower liver cancer risk. In the present study, we also further refined both the definition of liver cancer cases (to HCC only) and utilized more detailed data on physical activity (at four points in time and focused on moderate‐intensity to vigorous‐intensity leisure time activity).

As the liver plays an active role in glucose and insulin signaling, physical activity may directly affect the liver through positively impacting these pathways. Hypothesized mechanisms thus include pathways related to insulin resistance, inflammation, and reduction in body fat 25. Physical activity improves insulin resistance and reduces risk of diabetes 26. In this study, diabetes may have been on the causal pathway between physical activity and liver cancer incidence, as estimates were attenuated after adjustment for diabetes. Physical activity also has been shown to reduce inflammation, another risk factor for cancer 27. Lastly, obesity has been shown to be a risk factor for liver cancer. Mechanisms are still not fully understood, but researchers hypothesize that adipose tissue, which is highly metabolically active, may release hormones, growth factors, and signaling molecules such as cytokines, TNF‐α, and IL‐6, which influence behavior of other cells 28.

Strengths of our study include the large, prospective nature of our cohort and reported physical activity levels at four distinct ages. Our data were collected from individuals who were healthy at baseline, such that differential recall is not an issue, and participants were followed up for disease outcomes over time. Limitations of our study include that physical activity was self‐reported and may have changed in the interval between the risk factor questionnaire and diagnosis. However, capturing trajectories may give a better picture of the effects of lifelong activity rather than the most recent levels. Still, physical activity was recalled by participants at the specified time intervals, and may have been misreported, and possibly differentially reported by BMI status. The categories of physical activity were also crude measures, with limited categorical choices for respondents, which may have limited more finely assessing physical activity levels. We were also unable to stratify by other participant characteristics due to decreasing cell sizes. Although we did not have information of hepatitis B and C infection, in a previous study in this cohort, authors examined whether frequency of physical activity was associated with hepatitis B and C virus infection status in NHANES 1999–2006 and found no evident patterns of association 11.

In conclusion, our study suggests that consistent participation in physical activity throughout the life course may be most relevant in relation to liver cancer incidence. Studies with more detailed information on physical activity at multiple points over the life course are needed to confirm this finding and to better describe whether there is a critical age beyond which one cannot reverse effects of an inactive lifestyle.

## Conflict of Interest

None declared.

## Supporting information


**Table S1.** Physical activity trajectories and liver cancer incidence sensitivity analyses.
**Table S2.** Physical activity trajectories and liver cancer incidence using a restricted ICD histology codes (8170–8175; *n* = 417 cases).
