# Supplementary material for: Physical activity across the lifespan and liver cancer incidence in the NIH‐AARP Diet and Health Study cohort
Source: Cancer Med. 2018 Mar 13;7(4):1450–7. doi: 10.1002/cam4.1343 (PMC5911600; doi:10.1002/cam4.1343)
Supplement: Supplementary file 1 — Table S1. Physical activity trajectories and liver cancer incidence sensitivity analyses. Table S2. Physical activity trajectories and liver cancer incidence using a restricted ICD histology codes (8170–8175; n = 417 cases). [file CAM4-7-1450-s001.docx]

**Supplemental Table 1. Physical activity trajectories and liver cancer incidence sensitivity analyses**

|  | **Referent** | **Trajectory 4** | **Trajectory 6** | **Trajectory 3** | **Trajectory 1** | **Trajectory 5** | **Trajectory 7** |
| --- | --- | --- | --- | --- | --- | --- | --- |
| HR (95% CI)^a^ | ***Maintainers*** |  |  | ***Increasers*** |  | ***Decreasers*** |  |
| Model a | 1.00 | 0.82 (0.60-1.11) | 0.65 (0.38-1.10) | 1.12 (0.74-1.68) | 0.91 (0.55-1.52) | 1.30 (0.93-1.81) | 1.16 (0.83-1.62) |
| Model b | 1.00 | 0.75 (0.56-1.01) | 0.69 (0.43-1.12) | 1.04 (0.70-1.54) | 0.76 (0.45-1.27) | 1.25 (0.90-1.73) | 1.10 (0.80-1.52) |
| Model c | 1.00 | 0.75 (0.57-0.98) | 0.63 (0.40-1.01) | 0.95 (0.65-1.39) | 0.75 (0.46-1.21) | 1.21 (0.90-1.63) | 1.14 (0.85-1.52) |
| Model d | 1.00 | 0.81 (0.62-1.06) | 0.72 (0.46-1.13) | 1.04 (0.72-1.50) | 0.84 (0.54-1.33) | 1.24 (0.94-1.65) | 1.12 (0.84-1.49) |
| Model e | 1.00 | 0.74 (0.56-0.97) | 0.66 (0.41-1.05) | 0.94 (0.64-1.38) | 0.74 (0.46-1.20) | 1.28 (0.95-1.72) | 1.18 (0.88-1.58) |

^a^ **Restricted to those with no history of diabetes (n=271,744).** Adjusted for sex, with age as the underlying time metric. Also adjusted for race (non-Hispanic white, Black, Other), coffee intake (non-drinkers, ≤1 c/day, 2-3 c/day, 4-5 c/day, 6+ c/day), alcohol intake (non-drinkers, ≤1 drink/day, 1-<3 drinks/day, ≥3 c/day), and smoking history (never, former, current).

^b^ **Restricted to those reporting excellent, very good, or good health at baseline (n=260,657).** Adjusted for sex, with age as the underlying time metric. Also adjusted for race (non-Hispanic white, Black, Other), coffee intake (non-drinkers, ≤1 c/day, 2-3 c/day, 4-5 c/day, 6+ c/day), alcohol intake (non-drinkers, ≤1 drink/day, 1-<3 drinks/day, ≥3 c/day), and smoking history (never, former, current).

^c^ Adjusted for sex, with age as the underlying time metric. Also adjusted for race (non-Hispanic white, Black, Other), coffee intake (non-drinkers, ≤1 c/day, 2-3 c/day, 4-5 c/day, 6+ c/day), alcohol intake (non-drinkers, ≤1 drink/day, 1-<3 drinks/day, ≥3 c/day), and smoking history (never, former, current), **as well as BMI reported at age 18.**

^d^ Adjusted for sex, with age as the underlying time metric. Also adjusted for race, coffee intake, alcohol intake, and smoking history, as well as **self-reported health status (excellent/ very good, good, fair/ poor).**

^e^ **Excluding those with <2 person years (n=287,914).** Adjusted for sex, with age as the underlying time metric. Also adjusted for race (non-Hispanic white, Black, Other), coffee intake (non-drinkers, ≤1 c/day, 2-3 c/day, 4-5 c/day, 6+ c/day), alcohol intake (non-drinkers, ≤1 drink/day, 1-<3 drinks/day, ≥3 c/day), and smoking history (never, former, current).

**Supplemental Table 2. Physical activity trajectories and liver cancer incidence using a restricted ICD histology codes (8170–8175) (n=417 cases)**

|  | **Referent** | **Trajectory 4** | **Trajectory 6** | **Trajectory 3** | **Trajectory 1** | **Trajectory 5** | **Trajectory 7** |
| --- | --- | --- | --- | --- | --- | --- | --- |
|  | ***Maintainers*** |  |  | ***Increasers*** |  | ***Decreasers*** |  |
| Model 2 | 1.00 | 0.79 (0.59-1.05) | 0.55 (0.32-0.94) | 1.04 (0.70-1.54) | 0.89 (0.55-1.46) | 1.42 (1.05-1.93) | 1.15 (0.84-1.58) |
| Model 3 | 1.00 | 0.86 (0.64-1.15) | 0.62 (0.36-1.06) | 1.18 (0.79-1.76) | 0.96 (0.59-1.57) | 1.32 (0.97-1.79) | 1.11 (0.81-1.52) |

**Model 2** Adjusted for sex, with age as the underlying time metric. Also adjusted for race, coffee intake, alcohol intake, smoking history, and (no diabetes or BMI.)

**Model 3** Adjusted for sex, with age as the underlying time metric. Also adjusted for race, coffee intake, alcohol intake, race, smoking history, diabetes, and BMI.
